# The TimeUse+ data set: 4 weeks of time use and expenditure data based on GPS tracks

**DOI:** 10.1007/s11116-024-10517-1

**Published:** 2024-08-07

**Authors:** Caroline Winkler, Adrian Meister, Kay W. Axhausen

**Affiliations:** Institute for Transport Planning and Systems ETH Zürich, 8093 Zürich, Switzerland

**Keywords:** Smartphone diary study, GPS tracking, Travel behavior, Time use, Household expenditures

## Abstract

This data paper introduces the TimeUse+ data set and outlines the comprehensive survey methodologies employed in its collection. The TimeUse+ study comprised two online questionnaires and a smartphone-based diary, recording travel, time use, and expenditure data. Participants were instructed to utilize the TimeUse+ application over a 4-week period. The app passively tracked participants’ movements via GPS and enabled them to annotate their trips and time spent at fixed locations with relevant time use and expenditure information. We begin by providing an overview of extant research on smartphone-based activity-travel diaries and the survey methodological research that informed the TimeUse+ app’s design. We then delineate the study design and components of TimeUse+, highlighting insights derived from prior focus groups and pretests. The data analyzed in this study were collected during the main study wave of TimeUse+, conducted in German-speaking Switzerland from July 2022 to February 2023. Approximately 63,000 individuals were invited to participate, of which approximately 10% initially indicated interest. Despite the study’s extensive and burdensome nature, we observed a net response rate of 2.1%. We present findings related to travel behavior, time use patterns, and expenditure habits, and discuss the challenges faced and lessons learned during each stage of development and execution of the TimeUse+ study. These extensive longitudinal data, which include validated information on travel mode and purpose, as well as detailed data on duration, social partners, and expenditures associated with activities performed at each destination, are being made available for further research.

## Introduction

In travel behavior research, activity-based approaches have been adopted for decades, stemming from the foundational work of Hägerstrand and Chapin, who argued that a focus on individual trips alone provides insufficient insight into the underlying motivations and contexts of a person’s travel patterns (Hägerstrand 1970; Chapin 1974). Already then, it was evident that individuals’ trips are not planned or performed in isolation, but are rather embedded in a complex activity schedule. Travel decisions are nested within a complex context, where factors such as job and household responsibilities, the distances that have to be traveled to reach the destination where a given activity is taking place, and the amount of money one has to spend are all juggled within the constraint of time. Thus, travel diaries commonly used to collect individuals’ activity participation and socio-demographic background data are designed to understand the complexity of time, space, money, and social interactions that drive travel.

Many governments conduct their own national surveys to better understand population-level trends. In Switzerland, the Federal Statistical Office oversees two such studies: the Household Budget Survey (HBS), a continuous study that incorporates approximately 250 new households each month to document their purchases (BFS: Haushaltsbudgeterhebung 2015-2017 2022), and the Swiss Mobility and Transport Microcensus (MTMC), a telephone interview that captures the travel behavior of a representative sample of 56,000 individuals for a single day, including trip purposes (BFS, ARE: Mobilitätsverhalten der bevölkerung 2023). The HBS tracks how different segments of the population spend and save money from year to year. The MTMC is advantageous for addressing questions that require a representative cross-section of the population for a single day, but cannot give a full, disaggregated picture of the context or choice situation in which the reported trips were made. Apart from similar transport-related studies, most European countries conduct national time use surveys following Eurostat guidelines to ensure data harmonization across nations. During participation in such studies, participants record their activities in 10-min intervals for one weekday and one weekend day, detailing secondary activities, locations, and social partners (Eurostat: Harmonised European Time 2018). Notably, Switzerland does not administer a national time use survey; however FORS, the Swiss Centre of Expertise in the Social Sciences, conducts the Swiss Household Panel yearly that collects metrics similar to that of a traditional time use survey using a telephone interview (Voorpostel et al. 2015).

Furthermore, activity-travel and expenditure information are especially relevant for estimating value of travel time metrics that comprise a majority of user benefits in cost-benefit analyses for transportation infrastructure investments, as well as for travel demand forecasting. Historically, such models are concerned with peak times associated with typical work patterns. Work has long been seen as an activity that structures a person’s day, in contrast to leisure that is typically found to have much more spatial and temporal variability (Schlich et al. 2004) and to be motivated by the people these activities are performed with (Stauffacher et al. 2005). The COVID-19 pandemic has caused a shift is this respect, as work has become more flexible in terms of when and where it is conducted (Currie et al. 2021). Also, Vega Gonzalo and colleagues (2023) used data from an EU-wide survey conducted in 2021 and identified effects of socio-economic characteristics on mobility habits, including unfortunate shifts towards traveling with a car. These changes in these major determinants of travel demand suggest the reestimation and recalibration of these models using recent data so that policy makers can make informed decisions when it comes to transport-related investments, land use allocation, as well as other social policy. Additionally, worsening climate conditions are enough to motivate the investigation of what motivates travel decisions and how and who can be nudged toward more ecologically-friendly forms of travel.

Travel diaries have gained widespread adoption as a tool for collecting comprehensive trip-level data enriched with the overall duration and cost of the trip, activities performed at the destination and during the trip itself that determine trip purpose, along with socio-demographic characteristics. The evolution of these diaries has progressively shifted toward passive data collection using GPS, as the traditional face-to-face interview, telephone interview, or paper-and-pencil formats have been found to be susceptible to biases like retrospective bias, under-reporting of trips, and rounding of start and end times (e.g., Madre et al. (2007); Stopher et al. (2007); Wolf et al. (2003)). GPS loggers, devices that are given to participants to carry around as they go about their day, were initially used to document daily travel behavior (e.g. Stopher et al. (2007)).

The smartphone has since emerged as a prominent data collection instrument, owing to its ubiquitous presence in individuals’ lives (especially when outside of the home; 92% of adults in Switzerland own a smartphone, 97% of whom use it every day (Deloitte: Global mobile consumer survey 2019)). Although the incorporation of passive logging with a smartphone into travel diary studies has been around for more than 10 years, the true potential of using smartphones as a data collection tool is arguably only beginning. Concerns around extensive battery consumption and GPS sensors and their coverage have been at the forefront of this discussion (e.g. Jariyasunant et al. (2012)). Today, though quality of phone battery life and GPS sensors may not be drastically better, smartphones themselves have more and more sensors^1^ that developers can take advantage of when programming smartphone apps, which can make a considerable difference (e.g. by only running the app when the phone is in motion). Still then, it may be that discontinuity in signal or technical problems related to the smartphone and the operating system it runs leads to missing observations (Struminskaya et al. 2020; Bähr et al. 2022).

Moreover, studies that solely collect passive GPS data lack contextual information. Even trip purpose and location types must be imputed for studies that do not require participants to validate their detected GPS tracks (e.g. Gao et al. (2021) for the MOBIS data set). To balance this, recent smartphone travel diary studies collect additional information, either within the app itself or using online materials. Alho and colleagues (2022) integrated an active diary component to the Future Mobility Sensing app to collect information on daily expenditures over 7 days. Calastri and colleagues (Calastri et al. 2020) had participants use the rMove app for 2 weeks and augmented their GPS data with information on participant’s social networks and major life course events using a separate web-based survey. Recent smartphone studies are clearly aiming to collect richer information about travel choice situations and are moving toward multi-day data collection to get a fuller picture of individuals’ short- and long-term patterns. Consequently, the value of a well-executed large-scale longitudinal travel, time use, and expenditure diary study is multifaceted. However, there is a delicate balance between the need to collect extensive data and the imperative to keep participant response burden low, a challenge that becomes more pronounced in the context of longitudinal studies where response burden compounds with each additional day of study participation.

The TimeUse+ study presented in this paper was developed and conducted in order to collect these activity-travel and expenditure data over 4 weeks using a smartphone diary app that used passive GPS tracking and an active diary component. The study as a whole was tested with over 200 individuals in spring 2022. The few adjustments made after the pretest yielded the final study configuration, which is the focus of this paper. Just over 63,000 individuals in German-speaking Switzerland were invited to participate using the TimeUse+ app for 4 weeks during this main study. All passively collected tracks and fixed locations were to be annotated with information on activities that were performed and for how long, whether anyone else was present, and how much money was spent (when relevant). For tracks, these activities are secondary activities during travel. Two online questionnaires collected information related to socio-economic characteristics, mobility tool ownership, and long-term expenditures, such as rent and insurance. In the end, over 1,300 individuals successfully completed the 4 week diary period and online questionnaires.

The remainder of this data paper is structured as follows: the Materials and Methods Section documents the entire study procedure and accompanying materials, including previous survey method configurations that were tested. The Results and Discussion Section provides insights on how the survey was deployed in a large-scale study, encompassing response rates at each stage of the study, as well as the socio-demographic characteristics of the final sample. Additional descriptive analyses of time use, travel, and expenditure behavior are presented in order to illustrate the full range of data collected using the novel survey method and to demonstrate applications of the dataset. The final Conclusion Section provides concluding remarks and suggestions for future studies.

## Materials and methods

**Fig. 1:**
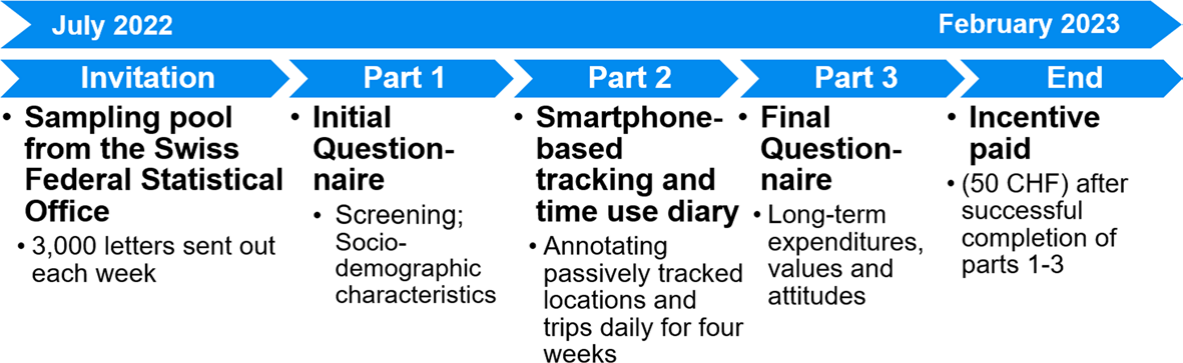
Overview of the TimeUse+ study design

Figure 1 presents a comprehensive schematic representation of the TimeUse+ study protocol. Data collection for the main study commenced in July 2022, with 3,000 potential participants receiving invitations on a weekly basis until December 2022. Given that participants were required to engage for a duration of 4 weeks, the study period extended through February 2023. The invitation delineated the three distinct phases of the study, encompassing an initial questionnaire, a 4-week tracking and validation period, and a concluding questionnaire upon completion of the tracking phase. The incentive payout of 50 Swiss Francs (CHF; $\approx$ 50 USD during the study period) was contingent on successful completion of all three study phases.

### Part 1: initial questionnaire

The questions included in the initial questionnaire reflect those commonly included as part of travel diary studies (e.g., Schmid et al. (2019); Aschauer et al. (2019)). This online questionnaire (hosted on Qualtrics) could be accessed via QR code or by entering the website printed on the invitation letter. The study information and requirements were first presented to participants along with a consent form and a privacy protection notice. Those who consented were then presented with a series of screening questions to eliminate individuals incapable of traveling 200 meters unassisted, professional drivers (e.g., taxi or bus drivers), or users of smartphones that were incompatible with the TimeUse+ app. The study was restricted to individuals aged 18 and above. The first block of questions encompassed socio-economic status, such as age, gender, education level, citizenship(s), and marital status. Subsequently, employment-related questions were posed, including current employment status, workload, workplace location, and remote work frequency. A third block inquired about participants’ mobility behavior and tool ownership, as well as household-level socio-demographic information, such as residential location and household size. Upon completion, participants were prompted to enter their email twice and were directed to the TimeUse+ website, which contained all pertinent study materials, including a *quick start* flyer (see Fig. 10) to help participants download the app and get tracking. The questionnaire took participants who indicated they wanted to participate 11.44 min to complete, on average (interquartile range = 7.43–13.10 min), and across everyone who at least started filling out this questionnaire 8.04 min (interquartile range = 2.78–6.87 min).

### Part 2: TimeUse+ smartphone application

**Fig. 2:**
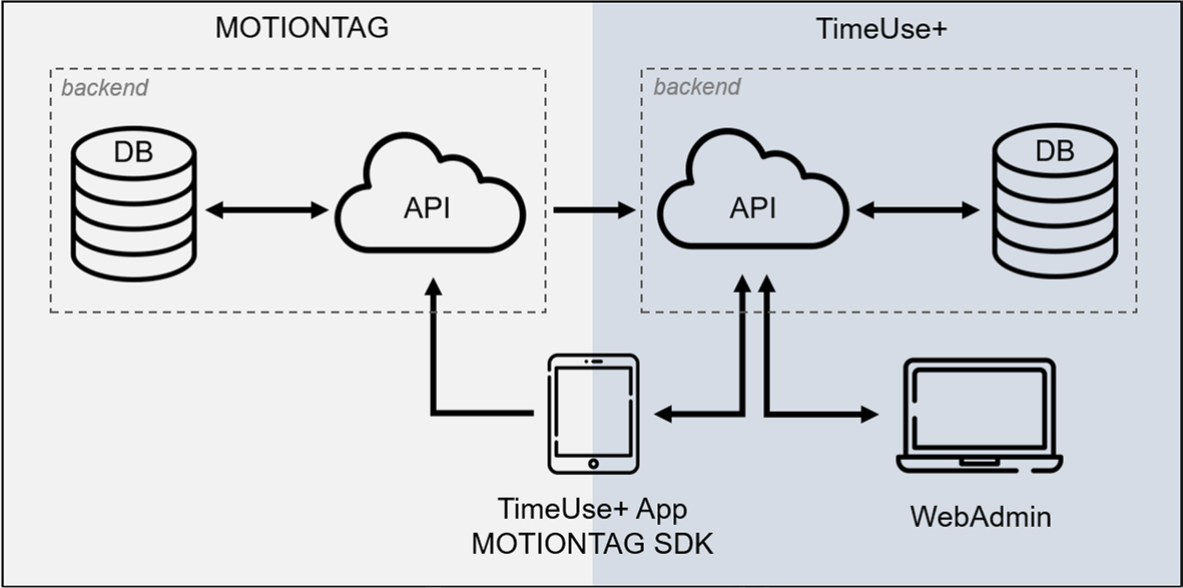
TimeUse+ platform architecture

The TimeUse+ platform comprises two primary components depicted in Fig. 2. The central component, the TimeUse+ app, facilitates participant documentation of their combined travel, time use, and expenditure diaries. The app incorporates MotionTag’s Software Development Kit (SDK) to transmit sensor data to the external MotionTag API, which subsequently performs trip and mode detection before conveying the results to the TimeUse+ backend. The diary format employs two distinct event types, stays and tracks, to populate a 24-hour timeline. These events can be augmented with additional activity and contextual data, such as social partners present and monetary expenditures.

The integrated diary format is assembled within the TimeUse+ API, merging the travel diary from MotionTag with self-reported time use and expenditure diary elements provided by users. This consolidated data stream is stored in a database and can be retrieved by the app for visualization purposes. Additionally, the app accesses a configuration file via the TimeUse+ API, which delineates various generic aspects of the survey format. Researchers can modify this configuration file through the WebAdmin interface. The platform enables researchers to customize event types, activity types, and activity attributes for a study via the WebAdmin interface, dynamically retrieved through the TimeUse+ API using the configuration file. This flexibility facilitates user experience by preventing unlikely combinations and allows researchers to explore specific research questions, such as online shopping during travel. Please refer to Meister et al. (2020) for a more detailed explanation of all technical components of the TimeUse+ platform.

**Fig. 3:**
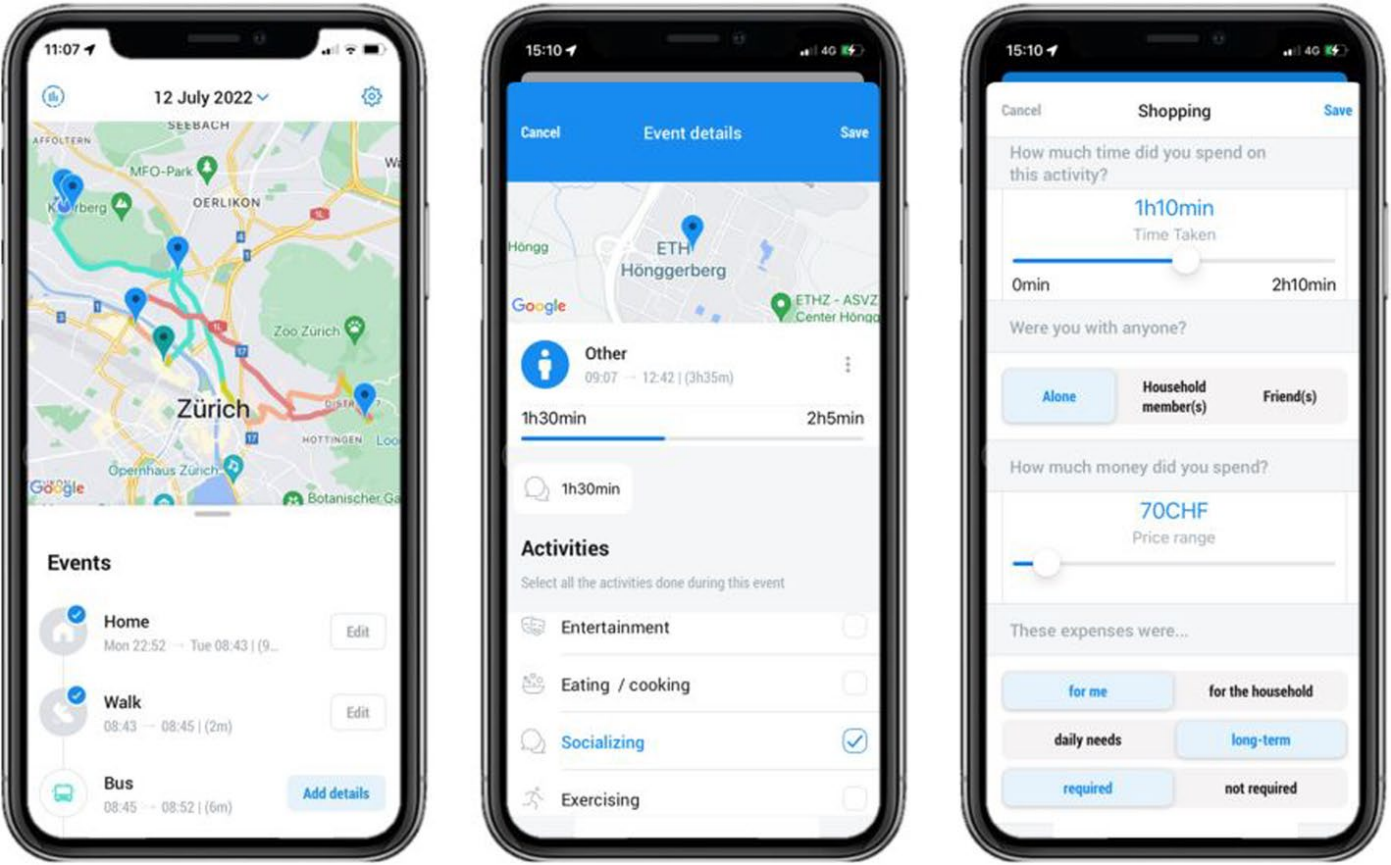
Screenshots from the TimeUse+ App. *Left:* Home screen, *Middle:* Event Details screen, *Right:* Activity Details screen

Figure 3 depicts screenshots of the TimeUse+ app. For any tracked day, participants can look at their timeline, which is accompanied by a map. The control bar that can be identified at the top of the leftmost screenshot offers access to the calendar view, statistics and settings screens (not shown). The calendar view denotes days requiring user validation, while the settings screen houses general settings, FAQ, and battery-saving options. The statistics screen summarizes weekly travel and time use behaviors, displaying frequently performed trips, activities, and associated CO emissions. The timeline differentiates between *stays* and *tracks*, the latter of which corresponds to *trip legs* or *stages* in the transport realm. The app ensures timeline continuity by inserting placeholder events in the case of missing tracks, which are labeled as *untracked*. The continuous timeline was deemed essential by individuals who participated in TimeUse+ focus groups, as opposed to having days that would start and end at midnight, which made reporting sleep and all other home activities confusing when switching between days in the timelines. In this final configuration, any event that stretched overnight was displayed and editable during both days that it took place.

The Event Details screen, illustrated in the middle of Fig. 3, permits users to correct an event’s detected mode or location, merge events, or delete events. A location validated as home or work is detected and displayed as such when participants revisit these places. The Activity Details screen, depicted on the right of Fig. 3, enables users to specify attributes like duration, social partners, and expenditures to each of the location and mode-dependent activities they perform during an event. Duration options are offered in 10-min intervals, while expenditure ranges are provided in 10 CHF intervals up to 200 CHF.^2^ Social partner and expenditure attribute options are fully customizable via the WebAdmin interface. To minimize participant burden, participants are asked for expenditure details only in relevant contexts, such as shopping or visiting a restaurant. Activities and all other aspects of the app were presented in either English, German, French, or Italian, depending on the language settings set on a participant’s phone.

For the main study wave, participants could choose from the following list of modes configured in the WebAdmin: airplane, bicycle, bike-sharing, boat, bus, cable car, car, carsharing, coach, e-bicycle, e-bike-sharing, kick scooter, motorbike/scooter, other track, car passenger, regional train, ski, subway, taxi/uber, train, tram, or walk. Of those modes, airplane, bicycle, car, light rail, regional train, subway, train, tram, and walk were automatically detected by MotionTag’s technology, and were otherwise labeled as unknown until validated by participants. Table 1 provides an overview of all activities that could be reported for each event type. Travel modes have been collapsed into broad categories that contain modes that shared the same activity options.

**Table 1:** Activity list by event type

Stay			Track			
Home	Work	Other	Car / motorbike (MIV)	Car passenger / public transport	(E-) bicycle	Walking
Sleeping	Working	Shopping	Nothing else	Nothing else	Nothing else	Nothing else
Self-care	Food break	Restaurant	Working	Socializing	Exercising	Socializing
Eating / Cooking	Socializing	Hobby / Leisure	Pick up / drop off	Eating / Drinking	Walking the dog	Eating / Drinking
Cleaning / Gardening	Digital entertainment	Eating / Cooking	Package pick up / drop off	Hobby / Leisure	Pick up / drop off	Hobby / Leisure
Socializing	Online shopping	Socializing		Digital entertainment	Package pick up / drop off	Working
Digital entertainment	Studying	Exercising		Working		Exercising
Hobby / Leisure	Other	Working		Resting		Walking the dog
Home-office		Studying		Online shopping		Pick up / drop off
Caretaking		Medical visit		Pick up / drop off		Package pick up / drop off
Online shopping		Caretaking		Package pick up / drop off		
Exercising		Waiting				
Resting		Pick up / drop off				
Studying		Package pick up / drop off				
Other		Sleeping				
		Resting				
		Self-care				
		Other				

MIV includes car and motorbike/scooter. Car passenger / public transport includes bus, regional train, train, subway, tram, car passenger, taxi/uber, and carsharing. The following changes were made in the first month of data collection, based on feedback received via email from participants: Gastronomy was renamed Restaurant, Entertainment was renamed Hobby / Leisure, General household work was renamed Cleaning / Gardening, and parking-related activities along with fueling as an activity for car were removed

### Part 3: final questionnaire

The second and final online questionnaire was automatically sent to participants via email upon successful tracking and validation for 4 weeks. This questionnaire captured long-term expenditures not reported through the app, including monthly rent, insurance payments, and subscriptions. Additionally, it featured personal statements rated on a seven-point Likert scale, addressing topics such as public transportation attitudes, environmental self-identity, and a short version of the BFI-10 personality inventory (Gerlitz and Schupp 2005). Participants provided their bank information within this questionnaire to receive the 50 CHF incentive. On average, completion of this questionnaire excluding the last IBAN question took participants 26.80 min.

### Insights gained from a pilot study and pretest

A pilot study performed in May 2021 served as a proof of concept of the TimeUse+ study as a whole. Thirty-five of 900 invited individuals completed the initial questionnaire, and 15 participated in all parts that were asked of them: a 1-week tracking period after the initial questionnaire, followed by a video call with the research team to discuss what worked well and what may need improvements. A 100 CHF incentive was provided. In short, we learned that:

1. We could expect a similarly low response rate, as is common in travel diary studies that use GPS (e.g., Calastri et al. (2020); Molloy et al. (2022); Stopher et al. (2018); Allström et al. (2017)).
2. The daily active involvement we were requiring from participants takes closer to 10 min than five to complete.
3. It was not unreasonable to expect people to document their daily expenditures (not too intrusive).
4. People participate because they find mobility studies intriguing or want to contribute to society, but rarely for the money they receive at the end.
5. The onboarding, usability, and the app as a whole was rated positively and there were no major issues.
6. Participants mentioned wanting to know more about why it was important that they participate and what their data would be used for.^3^ This last finding is in line with recent studies on willingness that stress that communicating the rationale to participants regarding why their GPS data will bring value to the project is critical for obtaining their consent in the first place (Struminskaya et al. 2021). In this regard, it is advantageous that GPS technology use is closely tied to mobility in everyday people’s lives and that ETH Zurich, the university running the study, is highly regarded among the Swiss general population. This background adds credibility to the motivation of running such a study.

In March and April 2022, a pretest was carried out to assess the feasibility of utilizing the TimeUse+ app for a 4 week period with a detailed activities list and a proposed monetary incentive of 50 CHF, rather than 100 CHF. Five distinct groups were established for this evaluation. Groups 1, 2, and 5 had a tracking and validating period of 4 weeks (while groups 3 and four participated for 2 weeks), Groups 1, 3, and 5 had a detailed activities list, and only Group 5 was provided an incentive of 100 CHF. To achieve this, two versions of the app were configured in the TimeUse+ WebAdmin for each of the two activity list types. The detailed activity list mirrored that used for the main study delineated in Table 1 with the "old" names referred to at the bottom of the table. The simple activities list used by Groups 3 and 4 was an aggregated version of the detailed list. For example, one could only choose between Sleeping, Self-care, Eating / Cooking, Chores, Leisure, Digital entertainment, Working / Studying, and Online shopping while at home.

The individuals invited to participate were randomly assigned to one of the five groups and the incentive they were to receive was clearly stated on their invitation letter. The study was successfully completed by 205 individuals, for an average net response rates across groups at around 2.73%.^4^ The pretest findings (see Table 5) indicated that reasonable response rates could be achieved even with a 50 CHF incentive for 4 weeks of participation. Participants who used the detailed list reported greater ease in documenting activities, as opposed to those using the simple list. Based on the experience gained during the pretest, we configured the detailed activity list in the WebAdmin for the main study and decided to offer participants an incentive of 50 CHF. The only change made to the app itself between the pretest and main study was an adjustment based on an iOS requirement from June 2022 to allow users to delete their data from within the app.

### Recruitment strategy and measures to combat attrition

The general procedure as outlined in Fig. 1 was run in 23 waves. Three thousand invitations were mailed each Monday; a total of 63,081 letters could be delivered. The invitation letter itself was carefully designed and formulated to convey trust, transparency, and professionality. The letter and mailing envelope included the logo of ETH Zurich, and its contents provided information introducing and motivating the study and participation requirements. Studies that investigate hypothetical willingness to participate in such surveys find that people are more willing to participate when the request comes from a university, compared to a marketing firm or statistical agency (Struminskaya et al. 2020; Keusch et al. 2019). The letter also mentioned that their address had been acquired by the Federal Statistical Office, which provides representative samples of the general population to a limited number of research endeavours that pursue ambitions that are of national interest each year.

During the seven-month study period, the research team implemented a systematic schedule to ensure prompt and continuous email correspondence with participants to sustain their participation. One team member spent 2 h responding to emails each day to handle the constant influx of emails (about 2,000 emails total over the seven-month study period). Apart from that email helpdesk, we employed an automated email system to maintain consistent engagement with participants and to provide them with timely updates on their status within the study. The first automated email was dispatched to participants upon the completion of the initial questionnaire. The content of this email varied based on whether the participant had already registered through the study’s mobile application. Every six days, an email was sent to participants who were not adequately validating their diaries, warning them that they would soon be disqualified and blocked from participating further.

The 50 CHF incentive served to signal appreciation for the time and effort involved in participation. Weekly personalized reports were also emailed (See Fig. 10), which provided interesting insights to participants about their travel patterns. These were intended to foster intrinsic motivation to continue participation, since participants in our focus groups assured us that they were not interested in the money, but rather interested in mobility patterns or research in general. Similar findings have been documented in the literature related to providing participant feedback. Indeed, when Wenz and colleagues tested the relationship between burden and satisfaction in participating in their study, 59.5% of participants given feedback reported their study participation as time "very well spent", while only 37.0% of participants did who did not receive feedback along the way (Wenz et al. 2022).

### Data preparation and analytical methods

Data from both questionnaires could easily be downloaded from Qualtrics using their API in R via the qualtRics package (Ginn et al. 2022). This "participants" data frame includes over 200 variables for 1,319 participants who each have one unique row and can be mapped onto the "activity-travel" data frame by participant ID. The activity-travel data were also pulled into an R workspace using the R package mongolite (Ooms 2014), which converts the JSON files from the MongoDB database that hosted the TimeUse+ data and creates R data frames. Data preparation included unnesting activities from events, disentangling merged events, adding placeholders for deleted data to keep the continuous timeline, imputing home locations and trips (elaborated upon below), verifying and correcting events’ time zones, and simplifying GPS points. The last step refers to assigning each event a start and end GPS point and an additional midpoint for track events such that each observation has some spatial information. The raw points are stored in separate data frames containing columns event ID, X GPS point coordinate, and Y GPS point coordinate. Further, some rounds of filtering based on start and end dates that had to manually be assigned for some participants were performed. Each observation in the final activity-travel data frame corresponds to a reported activity, which are all tied to the location or mode during which they took place by an event ID. The three mentioned data frames comprise the TimeUse+ data set.

Moreover, an issue that already arose during the pretest that could not be solved was that it was not always clear to participants that they should change the location type from *other* (default) to home or work. Most did, and the app labeled the location as such the next time they visited that location. For those who did not, however, we used DBSCAN in R (Hahsler et al. 2019), an unsupervised clustering algorithm, along with the activity information participants reported to impute home locations. The archived data set preserved the originally detected, validated, and imputed event names. The only other data processing applied to the data involved aggregating trip stages into trips in a first step and then to impute the main trip purpose by taking the activity performed for the longest time at the destination and the main trip mode based on the stage with the longest distance. After testing different thresholds, stages that were followed by a stay of 5 min or less and then followed by another stage were joined to form a single trip. A longer buffer of 20 min was used for stays between two public transport stages if it took place near a public transport stop (i.e., within 180 ms of stops by railroad operators or 20 ms of non-rail operators) and that *other* stay event had the activity waiting.

In general, the requisite data preparation is contingent upon the intended application of the data, specifically, the research questions it is meant to address. While travel behavior research mostly requires trip characteristics, time use analysis concerns itself with the activities performed at each location and during each trip. Due to the configuration of the app, only aggregated duration information and no timing information is available for different activities taking place during a stay event. Hence, if someone was home from 5 p.m. (Day 1)  until 8 a.m. the next day (Day 2), they likely ate dinner Day 1 and breakfast on Day 2. Participants recorded this behavior as one single "Eating / Cooking" activity and duration. Depending on the granularity of time use activities required for a research question, one may be content with having "at-home activities" as a single activity. Most time use analyses benefit from a more detailed list of activities and we therefore split at home events and activities at midnight. Several ways of splitting activities that were part of an overnight stay were tested. In the end, a proportional splitting solution was favored and applied for the time use analyses that follow. That is, if a person was at home seven hours on Day 1 and eight on Day 2, all activities were assigned to Day 1 based on their duration multiplied by 7/15 (47%). Another requirement of data for time use analysis is that all 1,440 min in a given day are accounted for and labeled. Gaps in the GPS tracks did arise, but were mostly hot starts before and in between trip legs. We chose to fill gaps according to prior work by Mesaric and colleagues (Mesaric et al. 2022). Both of these steps naturally come with implications that have to be kept in mind when using the data. Researchers who use these data in the future are encouraged to test different approaches for activity splitting and handling missing data.

In the activity-travel and expenditure analyses below, days with at least 20 h of information from 1,248 participants who have this data for 28 days (4 consecutive weeks) are included. The 71 participants who are additionally considered in the table of socio-demographic characteristics reflects individuals who are missing a day or two of data in between because of our lenience (required validation rate of only 70% that was unknown to participants). Data preparation requirements will vary from one research question to another, where 25 days of data will still be enough for the question at hand. This cleaned and filtered data set yielded 38,800 person-days.

## Results and discussion

### Response rates and attrition

The response burden score (Schmid and Axhausen 2019) for complete participation in all three parts of the study was conservatively calculated to be 3,600 points. This translates to 3,150 points for 30 days of tracking and validating (ca. 8.75 min per day, which is in line with anecdotal evidence) and another 450 points for both questionnaires for a grand total of 5 h of effort. The overall response rates are presented in Table 2. Approximately 56% of individuals who started the initial questionnaire were unable to proceed due to ineligibility. This can be partly attributed to the TimeUse+ app’s incompatibility with small-screen iOS devices or devices that are more than 10 years old. Among the 4,972 participants who reached the question about their smartphone type, 43 were disqualified due to not owning a smartphone or using a non-Android/non-iOS device. The remaining participants included 2,118 Android and 2811 iOS users. Nine-hundred-seventeen (33%) of said iOS users were disqualified because of device incompatibility due to small screen sizes.

Nearly all participants interested in fully participating in the study registered within the app. Hence, those who qualified seemed committed and were willing to provide their email and confirm interest during the initial questionnaire. Additionally, those who said they would participate and completed the initial questionnaire were able and eager to download the app and register without any issue. Roughly 73% of these registered participants commenced tracking, which implies a drop-out of nearly 1,000 individuals at this phase. Potential reasons for this attrition could be waning interest or initial curiosity that dissipated after acquainting oneself with the app’s features. Correspondence via email also revealed that some participants faced challenges configuring their phone settings to commence tracking. However, meticulous testing of the TimeUse+ app on various smartphone devices by the research team did not identify any operational issues with the application itself or its installation and registration process.

Of the participants who did begin tracking, 50% successfully completed the 30 day tracking and validation period. Ultimately, 1,329 individuals successfully finished all three TimeUse+ components and received their incentive payment. This corresponds to a net response rate of 2.11%, a seemingly low figure that is in fact comparable to smartphone travel diary studies that were not as burdensome (e.g. Calastri et al. (2020); Molloy et al. (2022); Stopher et al. (2018); Allström et al. (2017)).

**Table 2:** Response rates

Invited persons	*N*	63,081
Started initial questionnaire (Q1)	*N*	6856
	% of invited	10.87
Qualified	*N*	3859
	% of invited	6.12
	% of intro completed	56.29
Registered in survey	*N*	3749
	% of invited	5.94
	% of qualified	97.15
Registered in-app	*N*	3733
	% of invited	5.91
	% of registered in survey	99.57
Started tracking	*N*	2742
	% of invited	4.35
	% of qualified	73.45
Successfully completed tracking/validation period	*N*	1363
	% of invited	2.16
	% of qualified	35.32
	% of started tracking	49.71
Completed final questionnaire (Q2)	*N*	1329
	% of invited	2.11
	% of opened Q1	19.38
	% of qualified	34.43
	% of registered in survey	35.44
	% of registered in-app	35.60
	% of started tracking	48.47
	% of completed tracking	97.51

### Socio-demographic characteristics

Table 3 depicts the socio-demographic characteristics of participants of the TimeUse+ study compared to the national travel diary survey (MTMC) 2021, a representative sample of the Swiss population, filtered for adults in the German-speaking part of Switzerland where participants for TimeUse+ were recruited. A prior analysis investigated whether there were differences in the socio-demographic makeup of participants who dropped out along the way compared to that of the participants who made it to the very end considered here. No differences were found between the two groups. A few differences are observable, however, when the TimeUse+ sample is compared to the MTMC. Firstly, the TimeUse+ sample is skewed toward younger individuals, especially those between the ages of 18 and 40. The high employment rates, proportion of single individuals, and slightly higher monthly household income correlate with our young age bias. There is a stark difference in terms of education, with the TimeUse+ sample including a disproportionately high share of individuals who have a university degree. As far as mobility tool ownership is concerned, the TimeUse+ sample is unsurprisingly well-equipped with driving licenses and access to private motorized vehicles, but also public transport ticket subscriptions and bicycles. The bias toward university-educated individuals who are either highly mobile or are interested in mobility in general follows psychological principles of leverage-saliency theory and the mere-exposure effect. The former suggests that individuals’ interests will affect their propensity to participate in different types of studies (Groves et al. 2000), while the latter holds that individuals have an increased preference for things that they are more familiar with (Zajonc 1968). People who attend university have an idea of what research projects are and what they are used for, as opposed to their counterparts who may therefore perceive an invitation to participate in such a study suspicious in the first place.

**Table 3:** Socio-demographic characteristics of main study participants compared to MTMC 2021

		Main study	MTMC 2021
Characteristic		N = 1319	N = 4429
*Gender*			
	Women	627 (47.6%)	2281 (51.5%)
	Men	691 (52.4%)	2148 (48.5%)
*Age group*			
	18–40	521 (39.6%)	1082 (24.4%)
	41–55	459 (34.9%)	1155 (26.1%)
	56–65	221 (16.8%)	832 (18.8%)
	66 +	115 (8.8%)	1360 (30.7%)
*Citizenship*			
	Other	207 (15.7%)	636 (14.4%)
	Switzerland	1,111 (84.3%)	3,789 (85.6%)
*Education*			
	Low	26 (2.0%)	581 (13.2%)
	Medium	669 (50.8%)	2272 (51.6%)
	High	621 (47.2%)	1552 (35.2 %)
*Marital status*			
	Married	710 (54.0%)	2624 (59.3%)
	Single	462 (35.1%)	1069 (24.1%)
	Divorced	103 (7.8%)	430 (9.7%)
	Married, separated	19 (1.4%)	1 (0.0%)
	Widowed	13.0 (1.0%)	300 (6.8%)
	Civil partnership	9 (0.7%)	5 (0.1%)
*Occupation*			
	Employed or self-employed	1031 (80.0%)	2627 (59.3%)
	Retired	112 (8.7%)	1,306 (29.5%)
	Other	72 (5.6%)	374 (8.4%)
	Student	73 (5.7%)	122 (2.8%)
*Driving license*			
	Yes	1,243 (94.5%)	3721 (84.0%)
	No	76 (5.5%)	708 (16.0%)
Car access			
	No	103 (8.3%)	328 (8.7%)
	Sometimes	183 (14.7%)	749 (19.9 %)
	Yes	957 (77.0%)	2682 (71.4%)
	NA (no licesnse)	76	
*Season tickets*			
	GA	197 (15.0%)	513 (11.6%)
	Half-fare card	756 (57.4%)	1736 (39.2%)
	Other ticket type	73 (5.5%)	365 (8.2%)
	None	292 (22.2%)	1815 (41.0%)
*Bicycle access*			
	No	196 (14.9%)	1249 (29.0%)
	Sometimes	27 (2.1%)	346 (8.0%)
	Yes	1,093 (85.1%)	2715 (63.0%)
*Residential location area*			
	Urban	783 (59.8%)	660 (14.9%)
	Suburban	298 (22.8%)	842 (19.0%)
	Rural	229 (17.5%)	2,927 (66.1%)
	NA	9	-
*Monthly household income*			
	4,000 CHF or less	122 (9.3%)	610 (13.7%)
	4,001–8,000 CHF	418 (31.8%)	1455 (32.9%)
	8,001–12,000 CHF	418 (31.8%)	983 (22.2%)
	12,001–16,000 CHF	183 (13.9%)	403 (9.1%)
	More than 16,000 CHF	100 (7.6%)	280 (6.3%)
	Prefer not to say	75 (5.7%)	283 (6.4%)
	NA / Do not know	3	415 (9.4%)
*Household size*			
	1	199 (15.1%)	793 (17.9%)
	2	497 (37.7%)	1915 (43.2%)
	3	224 (17.0%)	636 (14.4%)
	4	307 (23.3%)	734 (16.6%)
	5 or more	92 (7.0%)	351 (7.9%)

### Travel data

To reiterate, travel behavior was passively recorded by the TimeUse+ app, and participants were asked to validate these tracks by correcting the mode, when necessary, and to add activity information for all events that were more than 5 min long. The stages and trips considered here all occurred in Switzerland.

#### Mode detection behavior

**Fig. 4:**
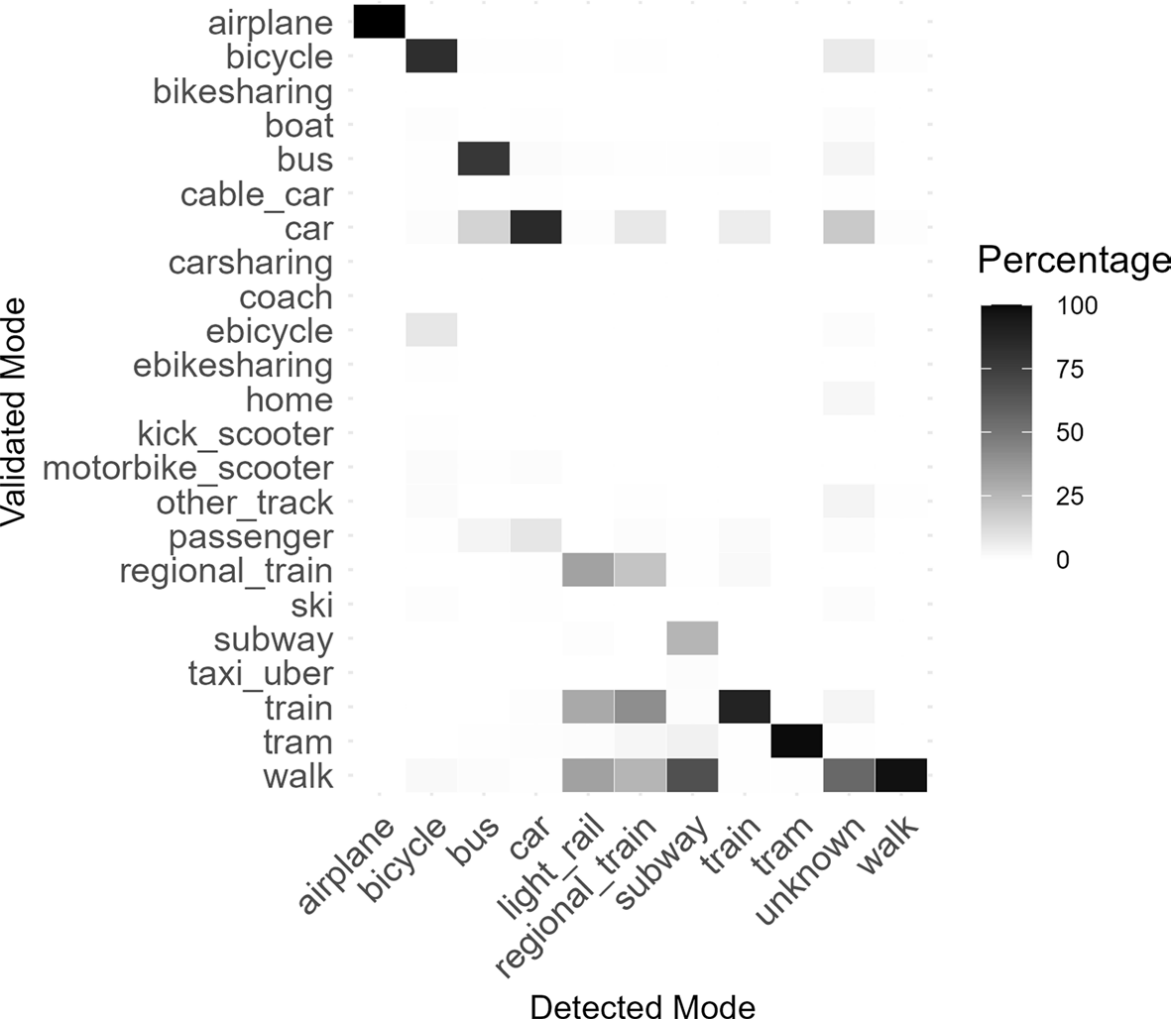
Heatmap of confusion matrix

A confusion matrix was computed to investigate the difference in detected versus validated travel modes. Because participants were required to validate their tracks, these differences can be interpreted as performance levels of the mode detection by modes. In other words, one would expect that all detected modes match almost perfectly (i.e. nearing 100%) with their corrected, or *validated* counterpart. For modes like car or bicycle, however, it makes sense that car passenger or taxi/uber or bicycle sharing requires personal corrections. As can be seen in Fig. 4, airplane, tram, and walk were never validated as being a different mode than detected. Bus, bicycle, and car were in a few instances corrected for reasonable alternatives. Unknown was validated as walk in 55.88% of cases, car in 18.63% of cases, and as other modes to a much lesser extent. Train was correctly detected 88.69% of the time, while regional trains, light rail, and subway were modes that were often changed. It is worth mentioning that no city in the sample area has a subway system. Kantons like Zurich, however, do have an urban-suburban rail network ("S-Bahn"), which is in fact neither a subway nor a light-rail. The S-Bahn can be used to quickly cross the city of Zurich underground, which could lead some people to see it as a type of "subway" or other rail network. The mismatch between the rail types is not concerning because of this level of subjectivity involved. Contrarily, as can be seen at the very bottom of the heatmap, the app seemed to detect different types of rail for trips that were actually performed on foot. It must be said that one could make this distinction because of the vast difference in speed between walk and rail services using the raw GPS points alone. Regardless, it is clear that requiring validation from participants improves data quality of detected travel behavior.

#### Key figures compared to MTMC 2021

We compared the unweighted TimeUse+ data to that published in the report of the 2021 MTMC, which was conducted from January 2021 until February 2022 (BFS, ARE: Mobilitätsverhalten der bevölkerung 2023). The statistics provided frequently reference data from the year 2015 or earlier for comparison. The authors provide a timeline of events and caution that values from 2021 may require careful interpretation due to the confounding influence of COVID-19-related restrictions that were still in place at various points in 2021.

Firstly, participants in our study recorded an average of 7.22 stages and 4.26 trips per day. These figures are lower than those obtained from the 205 participants in the TimeUse+ pretest, which stood at 7.98 and 5.56, respectively. However, they significantly exceed the data reported by the MTMC for 2021 (3.8 and 2.8) and 2015 (4.9 and 3.4). Such discrepancies are often noted when juxtaposing GPS-based studies with methods such as telephone interviews used by MTMC. Notably, GPS captures a large proportion of brief trips that are not remembered or mentioned during telephone interviews.

**Fig. 5:**
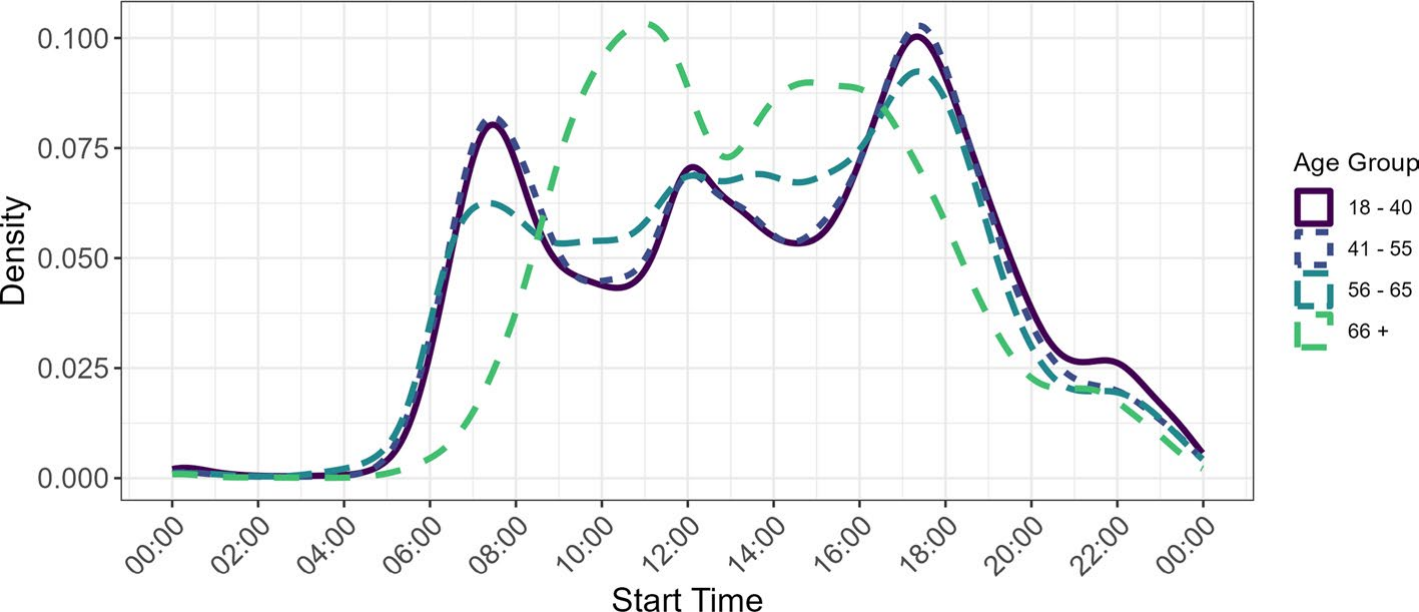
Stage start times on weekdays by age group

Another reason we find much higher rates is because the people in our sample are generally more mobile than the general public. Participants in the TimeUse+ study traveled on average 91.85 min per day and 45.65 kms per day (without wait times). These are higher than that reported by MTMC for 2021 74.6 min and 30.0 kms and 2015 82.2 min and 36.8 kms. We found both age and gender differences, with men traveling 51 kms per day compared to 41 traveled by women. We also compared daily distance traveled across four age groups: 18–40, 41–55, 56–65, and over 66. On average, they traveled 46, 47, 48, and 40 kms per day, respectively. Figure 5 depicts the density of trip leg start times for weekdays for these age groups. There is a clear distinction between participants 55 and younger, who have the the expected peaks that are related to commute times, while those in the oldest age group who are likely retired tend to move around between 9 a.m. and 6 p.m..

**Fig. 6:**
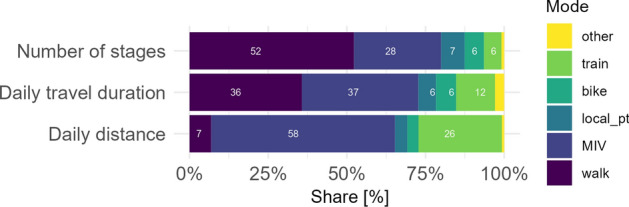
Mode choice for stages

The TimeUse+ sample is also more reliant on public transportation than the Swiss population as a whole. The mode shares were computed for a collapsed list of modes, as presented in Fig. 6. For car (MIV) alone, the 2021 rates reported in the MTMC are as follows: 69% of daily distance (compared to our 58%), 39% of daily travel duration (vs. 37%), and overall 37% of all stages (vs. 28%).

**Fig. 7:**
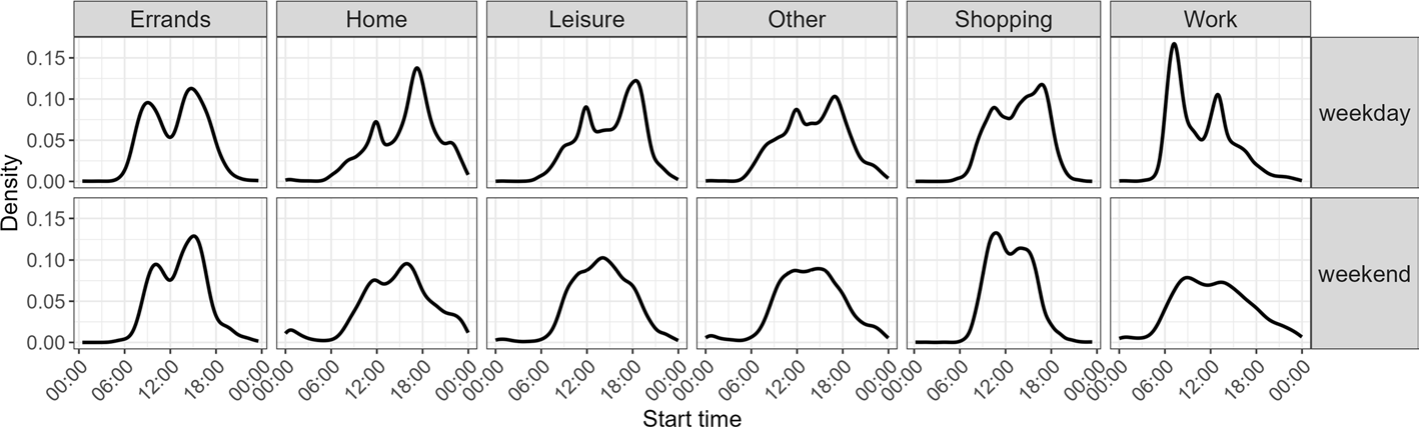
Start time by trip purpose

After grouping stages into trips, we plotted a density plot to show the distribution of start times for different purposes for weekdays and weekends. Trip purposes were aggregated into six categories: errands, home, leisure, other, shopping, and work. All activities that are not home were performed outside of the home. Figure 7 shows expected start times, such as work starting in the morning and for some also after lunch, while participants typically took a trip back home around 5 p.m.. Leisure activities start in the late morning and in the evening on weekdays and are distributed throughout the day on weekends.

### Time use

**Fig. 8:**
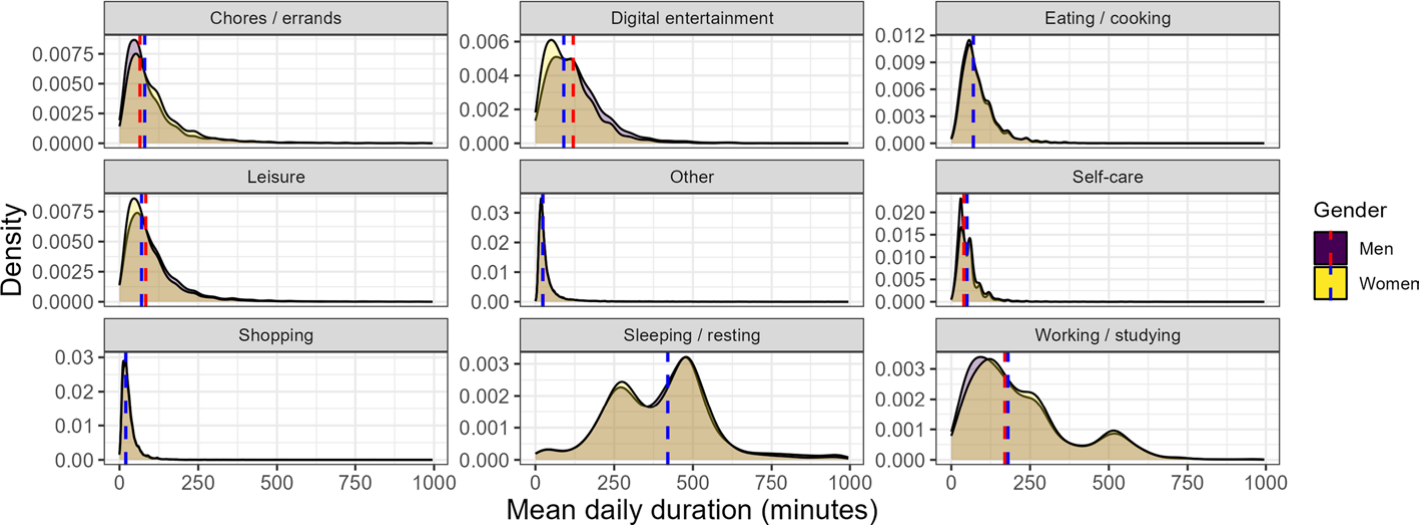
Average daily activity duration in minutes per person

The rich time use information collected with the TimeUse+ app stems from the stay events, rather than the trips that make up a mere 6% of one’s day. As alluded to earlier, activity lists were location-dependent and varied from a eight possible activities at a workplace to 16 different categories at location other. These data cannot be compared, as there is no national time use survey in Switzerland and the last available data from the German Time Use Survey are from its 2012/2013 wave. The decade in between has included shifts in time use for Europeans in terms of increased screen time, including to connect on a personal and professional level with others, flexible work locations and an uptick in the gig-economy, as well as online shopping, education, and counseling. Figure 8 illustrates the distribution of the average amount of time spent each day on different types of activities for men and women. The graph uses vertical lines to indicate median values for each of the genders. It can be seen that men spent slightly more time than women on activities such as leisure and digital leisure, while women in our sample generally spent more time each day on Chores / Errands and Self-care. Overall, our data show that participants spent close to 7 h sleeping each night, another 3 h and 45 min working each day, almost two hours participating in leisure activities, and 30 min shopping. Both genders spent a similar amount of time on sleeping, shopping, eating, and other activities. This aggregated view is not practical for most research questions, as both weekdays and weekend days are included, as are retired, part-time, and non-working individuals. Nevertheless, these findings appear plausible, underscoring the validity of our data set and survey tool, even when investigating the 40,320 min available over a span of four weeks.

#### Working from home

As the data were collected in 2022, following the major waves of the COVID-19 pandemic, this timing provided an opportunity to gain valuable insights into remote working arrangements and related preferences. The following descriptive analysis was performed based on a subset of 973 individuals who were identified as either employed or self-employed, out of the sample of 1,248 individuals included in the analyses described above. We used the complete month of tracking data for each individual participant. The initial questionnaire included specific inquiries about participants’ working circumstances, and included questions about whether participants were permitted to work from home and their current employment status. In Switzerland, employment contracts commonly specify workload in terms of percentages. In this context, a 100% contract corresponds to a total of 164 working hours per month, calculated as 41 h per week. Participants reported their workloads in the initial questionnaire, and for the purposes of analysis, these workloads were categorized into three groups: up to three days, 3–4 days, and four and a half to five days of work per week. Based on the upper bounds of these categories, expected average monthly working hours were estimated to be approximately 98, 131, and 164 h, respectively. It is important to note that a person with a 60% contract or working up to 98 h per month does not necessarily work three full days, but could have more flexible arrangements. According to the working hours reported in the diary components of the TimeUse+ app, participants who reported working up to three days per week averaged 83.71 h; those working 3–4 days averaged 124.17 h; and those working four and a half to five days averaged 149.02 h. These reported figures closely align with the expected number of working hours based on participants’ contractual obligations. It is worth noting that the observed average reported hours are slightly lower than the expected values. Possible explanations for this discrepancy could include participants working during their commutes and not accounting for these hours, or participants excluding break times from their reported working hours, among other factors. It may also simply be the nature of this real-life data, which means that participants could have been tracking during their vacations or days off.

**Fig. 9:**
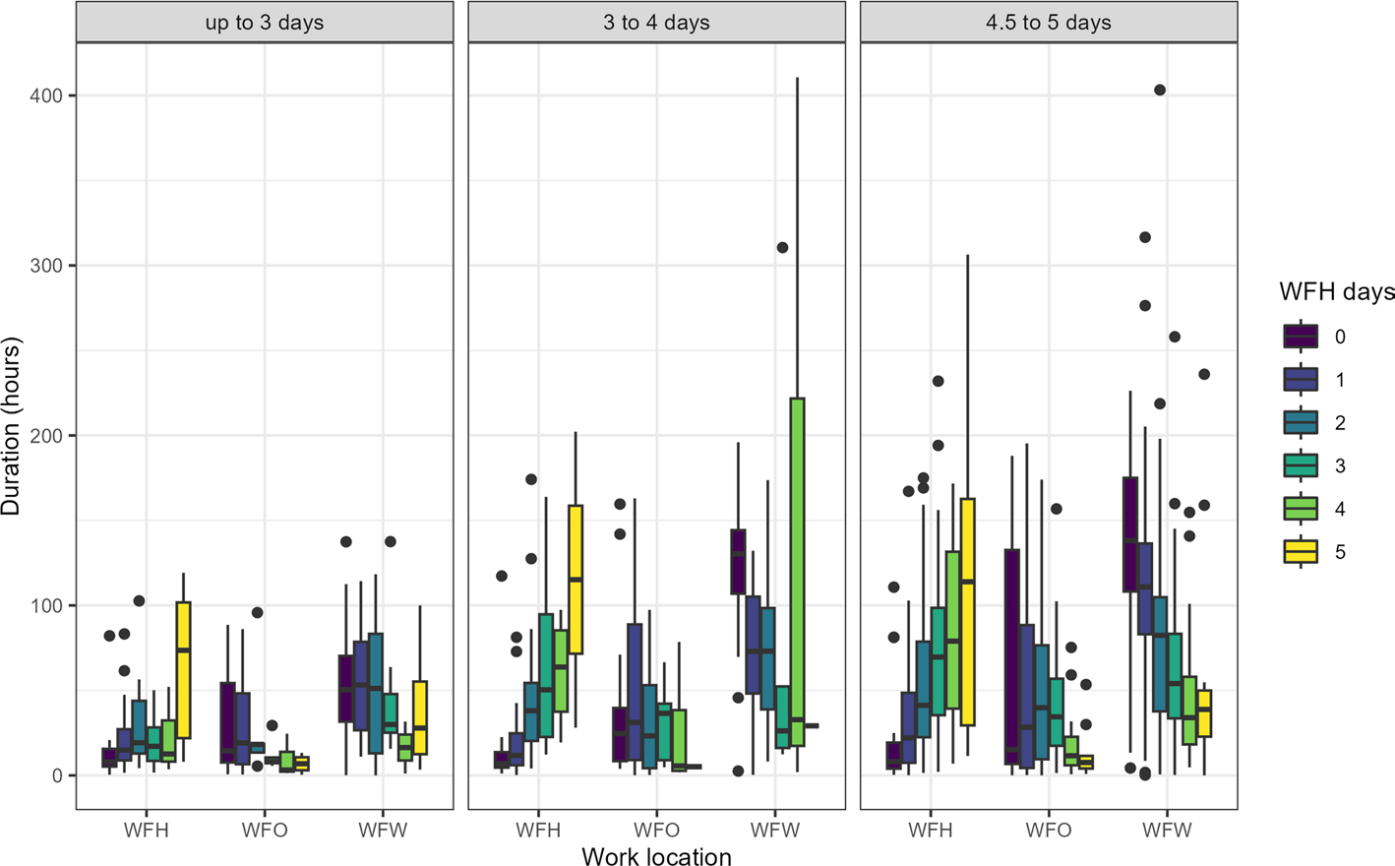
Average number of hours worked per month by work location and number of days one works from home

**Fig. 10:**
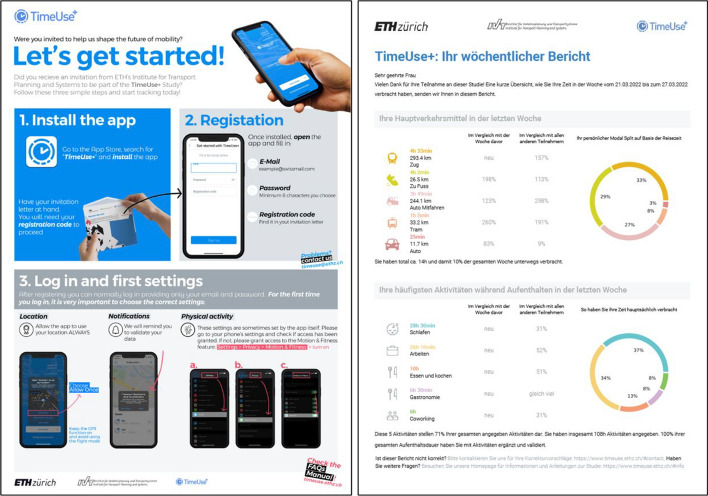
*Left:* Quick Start flyer for participants who successfully completed the initial questionnaire, *Right:* Sample personalized weekly report

Figure 9 provides a visual representation of the variability inherent in participants’ work lives, highlighting the real-life nature of the data collected. For this analysis, only participants who indicated that they were permitted to work from home were included, amounting to 533 workers, or 54.78% of the relevant subset. The x-axis of Fig. 9 delineates the work location, categorizing it as: Working from Home (WFH), Working from Workplace (WFW), and Work conducted at other locations that are neither home nor workplace (WFO). The y-axis depicts the average amount of hours worked over the entire month. The colors within the figure correspond to how many days per week participants reported that they typically work from home in a given week in the initial questionnaire. The right panel of Figure 1 focuses on full-time employees. It is evident from the figure that participants’ behavior is generally consistent with the WFH proportions they reported. However, it is important to highlight that participants who reported typically not working from home at all (0 days), as well as those who indicated that they usually work from home every workday (5 days), still recorded work hours outside of their "usual" location. While this pattern remains apparent for participants with a 60–80% contract, the leftmost panel, which includes individuals working up to 60%, does not exhibit a similarly identifiable pattern. It should be noted that this latter category is relatively infrequent, and thereby its numbers are not as robust.

### Expenditures

Expenditure information collected as part of the TimeUse+ study can be categorized as short-term, which includes everyday items such as groceries, or long-term, which includes rent and insurance costs. Short-term expenditures were collected via the app during activities that were likely to involve expenditures, such as shopping or visiting restaurants, while long-term expenditures were addressed in the final questionnaire. Please note that these larger and longer term payments are generally done by (automated) bank transfers in Switzerland. To facilitate questionnaire completion, the range of money that could be spent on these items was offered in ranges. For example, expenditures on a monthly combined phone, TV, and internet plan (given that one has one) could be reported as costing: up to 30 CHF, between 31 and 70 CHF, between 71 and 100 CHF, between 101 and 150 CHF, or more than 150 CHF. For the following analysis, we only used the midpoint cost of each possible category. Average expenditures per person per week reported within the diary app were as follows: 118.81 CHF on shopping, 64.46 CHF at restaurants, 16.48 CHF on leisure, 14.67 CHF on other activities, 10.97 CHF on errands, and 6.69 CHF on exercising.

We will examine our sample in alignment with the categories delineated by the Swiss Household Budget Survey HBS and their reported 2020 data, which has been published online (BFS: Household income and expenditure 2020). Evaluating based on singular values rather than distribution comparisons constrains conclusive interpretations; however, a comparative analysis is still insightful. Table 4 shows this comparison, based on categories used by HBS. Most TimeUse+ data come from the final questionnaire, and do not balance (i.e., 1,000 are unaccounted for) because of the categorical nature of the questionnaire and a few spending categories that are unaccounted for.

**Table 4:** Comparison of spending and saving patterns in Switzerland

Budget Structure		HBS 2020	TimeUse+
Major category	Minor category	[CHF]	[CHF]
Gross income		9817	9103
Mandatory transfer expenses		−2867	−3663
Monetary transfer expenditure to other households		−161	*NA*
Disposable income		6789	6651
Other insurance, fees, and transfers		−525	−573
Consumer spending		−4564	−3588
	Food and non-alcoholic beverages	−641	−484
	Alcoholic beverages and tobacco products	−102	*NA*
	Restaurants and accommodation	−343	−179
	Clothing and footwear	−138	−59
	Housing and energy	−1411	−1572
	Furnishings and current housekeeping	−207	−97
	Health expenditure	−237	*NA*
	Transportation	−630	−600
	Communications	−175	−124
	Entertainment, recreation, and culture	−394	−254
	Other goods and services	−286	−313
Sporadic income		182	*NA*
Savings amount		1881	1118

*NA* indicates that that category was not assessed in the TimeUse+ study. Each value stems from the final questionnaire except for Food and non-alcoholic beverages and Entertainment, recreation, and culture, which also includes average values from the diary app

Gross household income in the TimeUse+ sample is lower than that of HBS, likely due to the large amount of single-person households in our sample. On the other hand, mandatory transfer expenses like social security, taxes, and health insurance were reported as being slightly higher for individuals in our sample. The consumer spending categories cover everything from groceries, restaurant outings, and clothing to health expenditures, transportation, and different types of leisure and discretionary goods. The data from the app and long term expenditures combined illustrate these costs. In the end, HBS respondents spend around 4,500 CHF on these categories per month, compared to 3,600 CHF spent by TimeUse+ participants (including health expenditures not adequately covered in our study). This leaves HBS respondents with more money to save at the end of the month compared to our sample, 1,881 CHF and 1,118 CHF, respectively. While our expenditure data should be interpreted as an estimate, the results fall within a reasonable and plausible range. This indicates the validity of our data collection methods with respect to expenditure data.

## Challenges and lessons learned

Several challenges arose while designing and conducting the TimeUse+ study, which can be translated into learnings for future activity-travel and expenditure diary app efforts. A first general assessment is that such longitudinal studies require many person-hours of work, a resource consideration that cannot be overlooked. Two doctoral students worked on the TimeUse+ study since the beginning of 2020; that is, from deciding to have an app developed for the project until all data were collected. Both prior to and during the study, student assistants had to be recruited to help with the mounting levels of tasks. Two student assistants worked on major parts of the TimeUse+ project for up to 15 h each week since 2021. Though these positions were paid, this long-term commitment to the project was not ever guaranteed. Maintaining an open, collegial, and organized atmosphere as well as developing and implementing project management tools was pivotal for sustaining the engagement of the core TimeUse+ team. In pursuit of this, tasks were delegated to each member consistent with their respective interests, and weekly group meetings were convened to assess the output.

A third student assistant joined the TimeUse+ team in the summer of 2022 exclusively to aid with the helpdesk while the main study was running. As mentioned, this arduous task was crucial to keeping study participants committed to the project. The content of the emails received included everything from asking for more information about the study to complaining about certain aspects of the study. In fact, the three most common email subjects can be categorized as having to do with app complexity, participation burden, and issues with phone settings. Most of these were from participants who emailed us to drop out from the study. Participants had to contact us to stop receiving the automated emails (i.e., removing them from the backend on our side). Most settings-related issues stemmed from Android users. The research team meticulously documented the responses of specific Android devices when enabling tracking and notifications. However, given the diverse array of Android devices, each with unique settings configurations, it proved challenging to address every individual issue based solely on user descriptions, provided screenshots, and our firsthand experience from app testing and utilization.

The app complexity and burden issues are ones that can be dealt with in future projects. The complexity can be lessened depending on what the data are collected for. Travel diary data do not need to include a list of all activities done at a certain location, for instance, and may collect similarly useful trip purpose and trip mode information by solely requiring mode validation and reporting of a primary and secondary activity. Time use diaries, on the other hand, do require *at least* this granularity of data. Several challenges loom for time use diary smartphone studies, some of which can be counteracted with an optimized design. In our case, the TimeUse+ app could have been improved by changing the slider used to report expenditures and the way that users accessed the different stay and track types to correct them. Implementing such changes and then testing them across a range of phone types requires additional time and money, not to mention colleagues and friends who are willing to download a tracking app.

In general, our journey into smartphone app development for research purposes illuminated the complexities of collaborating with external app developers. The initiation of app development in 2020 posed challenges in sourcing developers receptive to a research-focused application without the promise of substantial financial gains, let alone the complications that arose due to uncertainties surrounding the COVID-19 pandemic. There is also a natural disadvantage in not having a tech specialist in a research team to evaluate the app’s code. Additionally, the sporadic updates demanded by operating systems emphasize the need for responsive development teams, which can be difficult in standalone projects without a committed team of developers to enact prompt adjustments.

## Conclusion

This paper introduced TimeUse+, an innovative activity-travel and expenditure diary study, offering a robust tool for gathering high-quality longitudinal data. This study serves as a significant addition to survey methodology, especially within the sphere of smartphone-based travel and time use diaries. Our research underscores the willingness of participants to engage actively in diary recording for an extended period of one month, demonstrating the feasibility of this approach. We contend that fully passive GPS diaries are not as valuable as ones that include validated information. To bolster participation and minimize attrition in future studies of a similar nature, researchers might consider initiating with a week of active engagement by participants, transitioning to passive data collection for the subsequent duration. A key advantage of this method is its ability to provide a comprehensive view of activities and expenditures at passively recorded locations. This mixing of subjective and objective survey methodologies to offset the disadvantages of each benefited both the research team and study participants. The study yielded a rich data set with confirmed modes and accurate trip and stay start and end times. On the side of the participants, there was no extra survey tool to carry around, and everywhere they had been on a given day was clearly depicted within the app, facilitating and lowering the burden of filling out the diary. While GPS’s early incorporation into time use research encountered hurdles, TimeUse+ showcases its efficacy, although it might fall short of the precision achieved by HETUS-based approaches due to the omission of detailed activity timing.

However, the TimeUse+ study is not devoid of limitations. Throughout the study, we encountered challenges in participant recruitment, retention, data processing, and app development, all of which are described in this paper, providing essential lessons for planning and budgeting future research projects in this field. A more seamless app functionality might have alleviated the ambiguity around why certain participants could not get started with the tracking process. This hiccup possibly led to a missed opportunity to increase our response rate. That the app’s design did not make it apparent to all that one must change the location other to home or work is an oversight that should be addressed in subsequent iterations of the study. Anecdotally, it was clear that participants did not use the supplementary study materials, such as the user’s manual where this was clearly described. In working with smartphone developers, it became evident that research-tailored smartphone applications occupy a niche domain. It is our hope that a stronger nexus between academics and developers will emerge in the near future, thereby expediting the advancement of this survey methodology.

Our diverse participant base of over 1,300 individuals revealed plausible patterns in travel, activity, and expenditure behaviors. Despite skewing towards a younger, more educated demographic, the compiled data present a rich resource for researchers across different disciplines. Addressing issues of representativity, strategies should be developed to effectively engage older demographics who are potentially less adept with smartphone technology. There is also a pressing need for community outreach to communicate the pivotal role of academic institutions and how study participation has the potential to influence policy, subsequently affecting the general population. Meanwhile, we invite the research community to leverage the data provided by TimeUse+, fostering a foundation for informed, data-driven policy and academic endeavors. Apart from further transport-related ventures, TimeUse+ data are well-suited for research in fields such as sociology, psychology, environmental science, geography, urban planning, economics, and public policy and are available via the ETH Research Collection (see Acknowledgements).

## Data Availability

The data for this study is being provided via the ETH Zurich Library Data Archive. Given the sensitive nature of the tracking data, we kindly request that interested parties contact the project team to engage in a discussion regarding access to this data. Data are available at the trip level, pseudo-anonymized and aggregated spatially in order to protect participant privacy. For those interested in working with higher resolution data, including specific routes and raw GPS tracks, please contact the research team directly.

## Appendix

**Table 5:** TimeUse+ Pretest: Recruitment and attrition

	Initial Q	Tracked	Completed	Final Q		% Final Q/Initial Q	% Invited/Completed
Group	M1	M2	M3	M4			
1	126	77	50	39		30.95	2.60
2	92	59	49	33		35.87	2.20
3	124	84	75	40		32.26	2.67
4	122	79	57	38		31.15	2.53
5	157	103	76	55		35.03	3.67
Sum	621	402	307	205	Mean	33.05	2.73

Group numbers correspond to the configurations presented in Table 1. M1-M4 refer to the milestones outlined in Figure 3. Q stands for questionnaire and Final Q reflects the final sample. Tracked individuals are those who began tracking using the TU+ app and Completed means that they reached the end of their required tracking period, but not necessarily that they validated the required 70% of events

## Footnotes

1 See Figure 1 in Struminskaya et al. (2020) for a timeline of the embedded smartphone sensors included in Android devices since 2010 when they only had six sensors, compared to 16 today.

2 Android devices allow for continuous expenditure choices.

3 It is possible that people who did not participate did not do so precisely because of such data privacy concerns.

4 See (Winkler et al. 2022) for a comprehensive account of the TimeUse+ pretest.
